# Development of the RIKEN database for dynamic facial expressions with multiple angles

**DOI:** 10.1038/s41598-023-49209-8

**Published:** 2023-12-08

**Authors:** Shushi Namba, Wataru Sato, Saori Namba, Hiroki Nomiya, Koh Shimokawa, Masaki Osumi

**Affiliations:** 1 RIKEN, Psychological Process Research Team, Guardian Robot Project, Kyoto, 6190288 Japan; 2 Department of Psychology, Hiroshima University, Hiroshima, 7398524 Japan; 3 Faculty of Information and Human Sciences, Kyoto Institute of Technology, Kyoto, 6068585 Japan; 4 KOHINATA Limited Liability Company, Osaka, 5560020 Japan

**Keywords:** Psychology, Human behaviour

## Abstract

The development of facial expressions with sensing information is progressing in multidisciplinary fields, such as psychology, affective computing, and cognitive science. Previous facial datasets have not simultaneously dealt with multiple theoretical views of emotion, individualized context, or multi-angle/depth information. We developed a new facial database (RIKEN facial expression database) that includes multiple theoretical views of emotions and expressers’ individualized events with multi-angle and depth information. The RIKEN facial expression database contains recordings of 48 Japanese participants captured using ten Kinect cameras at 25 events. This study identified several valence-related facial patterns and found them consistent with previous research investigating the coherence between facial movements and internal states. This database represents an advancement in developing a new sensing system, conducting psychological experiments, and understanding the complexity of emotional events.

## Introduction

Developing a new facial database will contribute to progress in many domains, such as psychology, affective computing, and cognitive science. Recent studies have reported on developing several facial databases well suited for many situations and research purposes, including deception detectione^1^, free speec^3^hes^2^, group discussion^2^, spontaneous tears^4^, pain-related face^5,6^, and social stigma^7^. The most enthusiastic among them has been the development of the facial expression database that conveys emotions (see the systematic survey^8^; review^9^; and meta-database^7^). These studies are the basis for extensive applied research^10,11^.

This paper introduces a new facial database called the RIKEN facial expression database with the potential to achieve multiple goals depending on the individual research aims for facial expressions of emotions. For example, the user can explore the relationship between facial movements and annotated information derived from the multiple theoretical views of emotion (emotional labels, valence, and appraisal components).

There are three issues in developing a new database for facial expressions of emotion: the deficit in multiple theoretical views of emotion, insufficient description of individualized events that induce affective responses, and the lack of multi-angle and depth information.

(a) In the scientific study of emotion, three overarching traditions can be identified: basic emotion theory, the theory of constructed emotion, and appraisal theory^12^. Each of these theories can distinguish itself by the psychological states they rely upon for emotional expression. Basic emotion theory involves emotional labels, the theory of constructed emotion encompasses valence and arousal values^13^, and appraisal theory centers on the appraisal dimensions of emotional events (novelty check)^14^. Many databases rely on basic emotion theory (i.e., six basic emotion categories^15–24^). Therefore, there is a lack of facial expression data based on other theoretical models of emotion. Available databases that apply the theory of constructed emotion are limited compared to those that apply the basic emotion theory or simplified emotional labels, and many existing databases sometimes use observers’ ratings of valence and arousal (AffectNet^25^; AFEW-VA^26^) rather than the expresser’s view (but see the Stanford Emotional Narratives Dataset^27^). Furthermore, the component process model (CPM^14^) derived from appraisal theory assumes that the results of each appraisal check drive the dynamics of emotion sequentially. The only facial database that relies on this is the actor database developed by ^28^. In the theoretical discussion of emotion, a recent scholar recommended the multiple ways to define emoion^29^. A database with annotations of various theoretical backgrounds would be desirable because it can be used flexibly according to the research purpose or practice.

(b) Contextual information is important to understand emotional expressions and perceptions^30–32^. Le Mau et al.^33^ emphasize the role of contextual information when inferring internal states through facial movements. Each emotional instance can be considered a loose concept^34^. Different persons perceive the same event differently. Being insulted may cause one person to feel anger while another may feel contempt or fear. An individual’s developmental history, including cultural learning, changes facial movements associated with affect^35,36^. Therefore, when expanding a facial database, it is important to address individualized eliciting contexts or conditions. A new database is expected to collect various perspectives on specific and personal events with several evaluations (labels, valence and arousal, or appraisal checks) rather than the same standardized situations.

(c) Many facial stimuli have been created using only two-dimensional (2D) images or clips^15,16,19–21,37,38^. Additionally, there are a limited number of facial databases with comparable multi-angle and depth information^28,39–41^. However, multi-angle data and depth information have several advantages in individual research practices. Psychologists have indicated that the angle of the face (e.g., frontal or profile view) is important in studying face perception. Guo and Shaw^42^ showed that profile faces have significantly decreased perceived intensity compared to frontal faces. If angles influence our facial perception, a database with multiple angles contributes to their psychological research. Moreover, multi-angle information is gaining attention in computer science. From multi-angle images, the state-of-the-art algorithm can generate volumetric radiance representation^43^. Echoing volumetric representation and 4D information, which adds dynamic information to 3D faces, have been the focus of increasing attention in psychology research, such as research on face perception^44^. For example, Chelnokova and Laeng^45^ showed that 3D faces could be recognized better than 2D faces. Scholars have developed new databases that directly measure depth information using tools such as Kinect to enrich science using facial databases^46,47^. Therefore, multi-angle recording and obtaining depth information have increasingly become standard in affective computing^28,48–50^. Multi-angle and depth information are expected to be useful in reconstructing a face with many features and extracting detailed facial movements. Collecting this information is important for conducting psychological experiments and training or developing automated sensing systems.

This study developed a new facial database that includes individualized contexts and multiple theoretical views of emotions with multi-angle and depth information. We aimed to create a facial database based on 25 individual events corresponding to valence and arousal^51^. Furthermore, we obtained free description labeling data^52^ and rating values associated with appraisal dimensions^53,54^ from the 25 events prepared by the participants. We are currently performing manual facial action coding using the Facial Action Coding System (FACS), a comprehensive, anatomically based system for describing all visually discernible facial movement^55^. This database makes these annotated facial movements publicly available data (8 people are already annotated and available). There are 29 types of manually annotated facial Action Units (AU). Please complete the following form (https://forms.gle/XMYiXaXHhfszCb4c6) to request access to the RIKEN facial expression database. People who want to use the RIKEN facial expression database must agree to the end-user license agreement.

Here, our main purpose was to report the characteristic of the RIKEN facial expression database. To understand the nature of emotional events within this database, we performed a text analysis of word frequency for all the events. Next, this study investigated the relationships between the appraisal dimensions, including valence and arousal, for the events. We then provided an overview of the characteristics of the events tagged with each emotion label. This was achieved by assessing the frequency of labels assigned by participants and examining the mean values of all the evaluative elements of the events. These can provide insight into the emotional events the database targets to create facial reactions.

Finally, we aimed to elucidate the nature of this database as a “facial” database. We quantified facial expressions using an automated FACS analysis and analyzed their annotated information, such as valence and arousal. This approach was designed to be a practical use case. As a well-established relationship, there is an association between AU 4 (brow lowerer) and negative valence, AU 12 (lip corner puller) and positive valence. We predicted these relationships because several psychophysiological studies recording facial electromyography showed that activity in the corrugator supercilii (related to AU 4) and zygomatic major muscle (related to AU 12) is negatively and positively associated with subjective valence experiences, respectively^56–61^. This study also conducted an exploratory investigation into the relationship underlying arousal and each facial muscle. The focus of this paper remains the demonstration of use cases.

## Methods

### Participants

Forty-eight Japanese adults (22 female and 26 male) aged between 20 and 30 years (mean = 23.33; SD = 3.65) participated in the recording sessions. The participants were recruited from a local human resource center in Kyoto. Individuals were informed about the purpose of the study, methodology, risks, right to withdraw, handling of individual information, and the voluntary nature of participation. All participants gave written informed consent before recording the facial movements. Informed consent included whether the participants agreed to their videos being shown for academic purposes, including psychological experiments and affective computing. Each participant was paid 13,000 JPY for participation and database creation. The Ethics Committee of the RIKEN (Protocol number: Wako3 2020-21) approved the experimental procedure and study protocol. The study was conducted according to the Declaration of Helsinki. Our main purpose was not to estimate population indices for effect sizes. Therefore, power analyses were not available.

### Procedures

**Figure 1:**
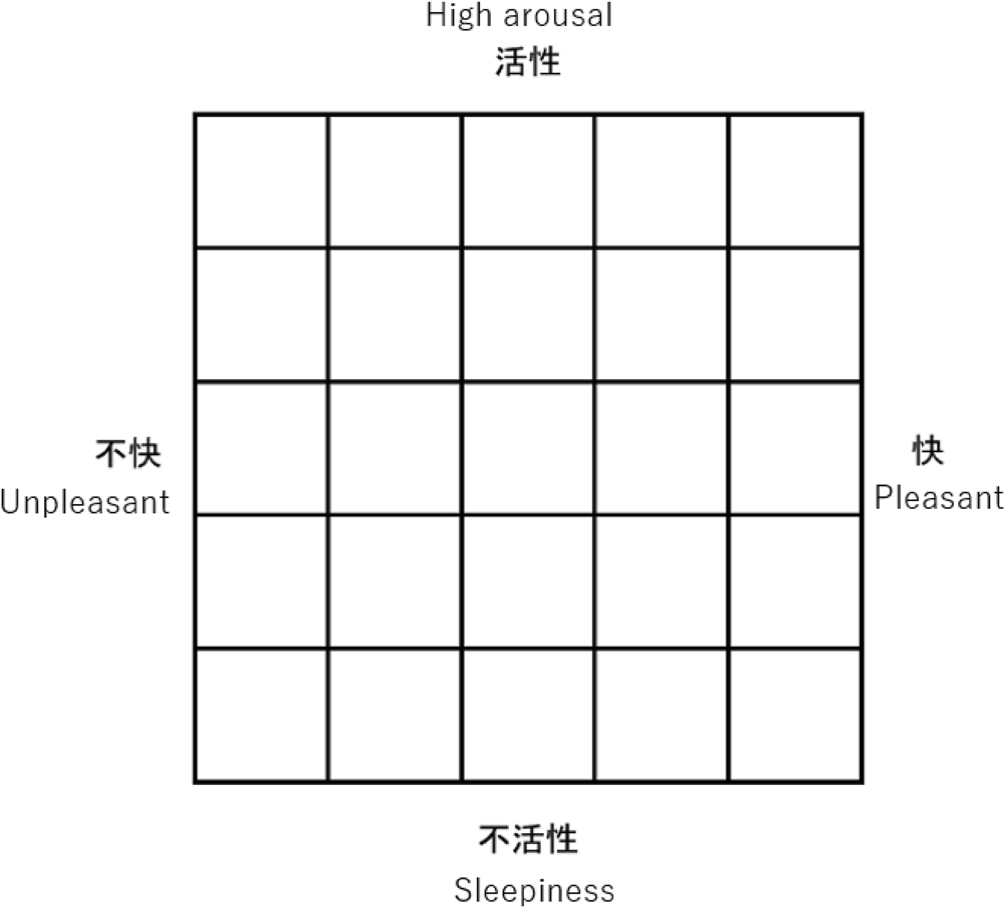
Events referred to the affect grid^51^.

All participants were instructed to remember and write down 25 events that occurred in their lives, with five valences (strongly unpleasant, unpleasant, neutral, pleasant, and strongly pleasant feelings) and five levels of arousal (very low arousal or sleepiness, low arousal, middle arousal, high arousal, and very high arousal) one week before the recording session to obtain individualized emotional events (Fig. 1). Qualtrics was applied as the platform for collecting the events. Participants were instructed to describe a single event corresponding to each cell in Fig. 1. The order of events (i.e., each cell in Fig. 1) for the valence and arousal combinations was randomized. Participants also rated appraisal checks from 1 (strongly disagree) to 5 (strongly agree) for novelty (predictable: “the event was predictable”; familiar: “the event was common”), goal significance (“the event was important to you”) and coping potential (“the event could have been controlled and avoided if you had taken appropriate actions”) for each event described. These appraisal checks were derived from a previously reported facial database that relied on the CPM as its theoretical basis^28^. The participants were also asked to describe the possible labels for each event freely.

**Figure 2:**
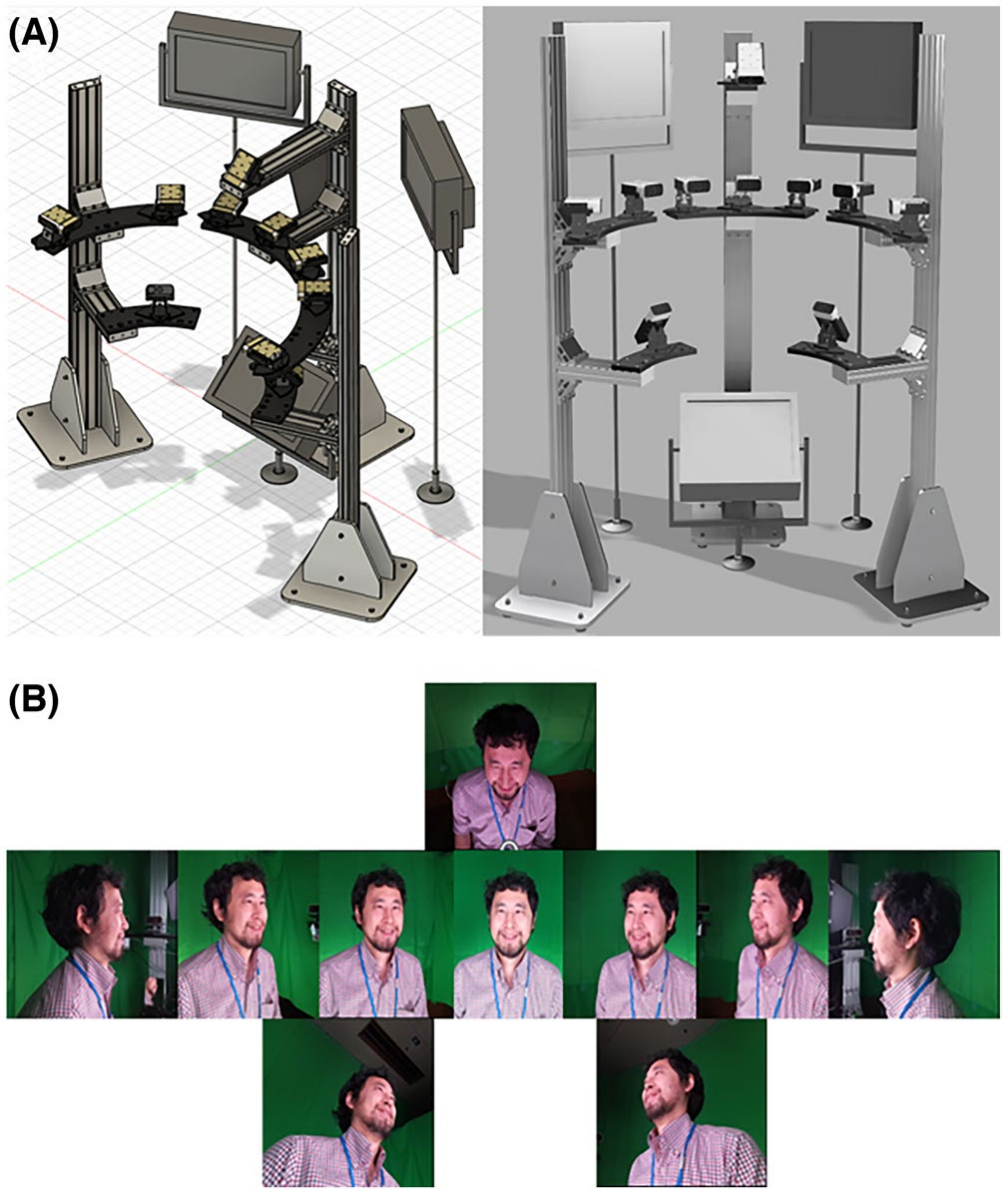
(**A**) The setup of the apparatus. The camera settings illustrated by Autodesk Fusion 360. Three lights were shone on the face from under the feet and from above on the left and right sides, and facial expressions were captured with one upper and two lower cameras, and front-facing, two left, and two right cameras. (**B**) Showcasing samples of the images. A green carpet covered as much of the background as possible.

On the day of the facial clip recording, the participants were given a further explanation of the experiment. They were transferred to the recording location (the first basement floor of the Advanced Telecommunications Research Institute International). Figure 2 displays the recording environment. The participants sat in chairs with their faces fixed in a steady position. We set up three photographic lights (AL-LED-SQA-W: Toshiba) and illuminated each participant’s face from the upper right, left, and lower sides to make clear their faces and remove shadows.

We then asked the participants to remove their masks and glasses. We set up an environment to record facial movements using ten Azure Kinect DK1880 cameras with (Microsoft; 2D:1920 × 1080; depth:640 × 576) pixel resolution and 30 frames per second to record the participants’ facial movements as video clips. The interval between the left and right horizontal cameras was 22.5°, and the cameras were used at 22.5°, 45°, and 90° (skipping 67.5° in this database). Images were taken to avoid interference between multiple depth cameras, with each camera shifted by 160 microseconds in timing. A Software Development Kit program was used to create a program to record facial movements. The depth information was limited to eight cameras to reduce the processing load and avoid equipment errors: one upper and two lower cameras, and front-facing, two left, and two right cameras; a green carpet covered as much of the background as possible (Fig. 2).

The experimenter verbally narrated the individual event descriptions collected one week before the recording session for each expression. Participants were instructed to vividly reexperience their emotions and practice expressing them through facial expressions using a hand mirror before the recording. Participants were allowed to remember the events and practice their facial expressions with no time restrictions. When participants felt ready, they sounded a bell to initiate the recording and the experimenter verbally narrated the events again. The recording process was structured into distinct segments. The timing of each segment was indicated by beep sounds (onset:880 Hz; peak:1174 Hz; offset:880 Hz) produced by the speaker system to control the participants in producing their expressions according to the time course. The models were instructed to express an emotional expression rooted in pre-described events for the initial 1 s, maintain the intended emotional expression for 2 s, and then return to a neutral expression for one second. The order of events was also randomized.

### Data analysis

We extracted 17 facial movements to evaluate the pattern of facial expressions using OpenFace^62^: AU 1 (inner brow raiser), AU 2 (outer brow raiser), AU 4 (brow lowerer), AU 5 (upper lid raiser), AU 6 (cheek raiser), AU 7 (lid tightener), AU 9 (nose wrinkler), AU 10 (upper lip raiser), AU 12 (lip corner puller), AU 14 (dimpler), AU 15 (lip corner depressor), AU 17 (chin raiser), AU 20 (lip stretcher), AU 23 (lip tightener), AU 25 (lips parts), AU 26 (jaw drop), and AU 45 (blink). Among the automated facial movement detection systems, OpenFace had a relatively good performance^63^. Since Namba, Sato, and Yoshikawa^64^ also found that facial images from the front view have the highest accuracy in OpenFace, only facial expressions from the front-view camera were targeted in this study. Given the procedure’s nature, in which facial combinations’ intensity is expected to be maximal during the apex beep sound, we mainly focused on the middle frame (i.e., 61 frames).

We used R^65^ for statistical analysis. We used the tm and openxlsx packages to perform text mining for each event^66–68^. The psych package^69^ was used to check the correlation between several appraisal dimensions. We used the nnTensor package to reduce dimension for data extracted by OpenFace^70^. We used the tidyverse package for data visualization^71^. Based on ample psychophysiological evidence, we predicted and analyzed the relationships between valence and AUs 4/12 using hierarchical linear regression modeling using the lmerTest package^72^. The results were considered significant at p < 0.05. To elucidate the relationship between arousal and AUs, we used Bayesian Lasso regression^73^, treating arousal as the dependent variable and utilizing all AUs as independent variables with the tuning parameter set at a degree of freedom of 1^74^. The AU data were standardized, and only results that did not encompass zero within the 95% confidence interval were reported. All codes are available on the Gakunin RDM (https://dmsgrdm.riken.jp:5000/uphvb/). The design and analysis of this study were not pre-registered.

### Ethics declarations

The Ethics Committee of the RIKEN (Protocol number: Wako3 2020-21) approved all experimental procedures and protocols. This research was conducted according to the Declaration of Helsinki.

### Consent to participate

All participants provided written informed consent to participate before the beginning of the experiment.

## Results

The detail of the events. As indicated in the "Methods" section, we obtained 1,200 events (48 participants × 5 valences × 5 arousals). All Japanese events were translated and back-translated into English using TEXT (https://www.text-edit.com/english-page/). Table 1 depicts the top 3 frequently used English words for each event obtained by text mining. In the obtained database, words that appeared to be common events (frequency of 10/48 or more) occurred in valence 4 * arousal 4 events (friend) and valence 5 * arousal 5 events (passing the university entrance exam). The latter, in particular, shows that university entrance exams greatly affected emotional events because this research was limited to young participants.

**Table 1:** Frequent words in each individualized event.

V1A1		V1A2		V1A3		V1A4		V1A5	
Sleep, exam	4	Sleep, wait	3	Person	9	Fail, bike	4	Someone	5
Bed, person	4	Small, get…	3	Dislike, got	4	Find, time	3	Person, hit	4
Depress, bad…	3	Tired, want…	2	Parttime, job	4	Bad, fell…	3	Fell, head…	3
V2A1		V2A2		V2A3		V2A4		V2A5	
Morning	7	Day, rain	5	Want	4	Work	7	Game	4
Sleep	6	Start, intend	3	Dislike, favorite	3	Take	4	Someone, make	3
Day, wake…	3	Something, well…	3	Well, hours…	3	Custom, train…	3	Mistake, get…	3
V3A1		V3A2		V3A3		V3A4		V3A5	
Watch, class	4	Train	5	Day, lunch	4	Take, time	4	News	5
Take, time	4	Ride, watch…	4	meal, without…	4	School, friend…	4	Day	4
Space, sleep	3	Bike, take…	3	Spend, time…	3	Room, make…	3	Time, watch…	3
V4A1		V4A2		V4A3		V4A4		V4A5	
Sleep	7	Good	6	Favorite, watch	5	Friend	11	Game	5
Bed	5	Watch	5	Buy, new	3	Favorite	6	Friend	4
Play, home	3	Holiday, movie…	4	Good, job…	3	Game, make…	4	Favorite, time…	3
V5A1		V5A2		V5A3		V5A4		V5A5	
Sleep	7	Message, won	4	Friend	7	Friend, favorite	7	Exam, pass	16
Work, hot	4	Finish, work…	4	Favorite	6	Play	6	Entrance	13
Holiday, coffee	4	Tire, good…	3	Time, watch	5	Watch	5	University	10

“…” means that there is more than one word with a frequency equivalent to the words listed in the table. V represents valence, and A represents arousal. The word ‘get/got’ was removed as it is used as a ‘be’ verb in Japanese.

The correspondence between the valence, arousal, and appraisal ratings is also presented in Table 2. Valence and arousal appeared to be positively associated with the appraisal of importance for each event (*r*s > 0.20). The results also revealed that high predictability increased the valence of the event (*r* = 0.22). Additionally, the more unfamiliar the event, the higher the arousal (*r* = −0.19). For the correlations between appraisal dimensions, positive correlations were found between predictability and familiarity, and predictability and controllability (*r*s > 0.29).

**Table 2:** Correspondence between valence, arousal, and appraisal ratings.

	Predict	Similarity	Important	Control
Similarity	0.451			
Important	0.147	−0.062		
Control	0.294	0.176	0.033	
Valence	0.215	0.079	0.237	−0.020
Arousal	−0.054	−0.194	0.208	−0.033

As the participants were asked to freely describe the possible labels for each event, each event had an emotional term that the participant subjectively labelled. To provide information labelled as an individual event, Table 3 lists the most frequently used labels of emotions using the free description data. Only the top 18 modes (N = 730/1200) are listed. Positive emotional labels, such as joy, happiness, and fun, indicated high valence; negative emotional labels, such as anger, sadness, and unpleasantness, indicated low valence. Arousal was high with surprise and impatience. Although there were other interesting correspondences between the controllability component and frustration, predictability, and fun, these were not examined as they went beyond the study’s purpose of overviewing the events in our database.

**Table 3:** Eighteen emotional labels and corresponding valence, arousal, and appraisal checks.

Emotional label	N	Valence	Arousal	Predict	Similarity	Important	Control
Joy (喜び)	119	4.45 (0.59)	3.21 (1.38)	2.83 (1.17)	2.92 (1.17)	3.91 (1.03)	2.99 (1.26)
Anger (怒り)	89	1.45 (0.52)	3.48 (1.39)	2.34 (1.15)	2.73 (1.20)	3.38 (1.25)	2.94 (1.34)
Happy (嬉しい)	59	4.46 (0.57)	3.51 (1.32)	3.03 (1.27)	2.69 (1.19)	3.93 (0.96)	2.37 (1.14)
Sadness (悲しみ)	54	1.43 (0.57)	3.02 (1.45)	2.54 (1.30)	2.56 (1.21)	3.70 (1.25)	3.15 (1.45)
None (無)	39	2.97 (0.35)	2.28 (1.07)	3.46 (1.41)	4.00 (1.08)	2.41 (1.23)	3.18 (1.41)
Relief (安心)	37	3.86 (0.75)	2.46 (1.24)	3.76 (0.93)	3.59 (1.19)	3.76 (0.93)	3.54 (1.04)
Fun (楽しい)	36	4.17 (0.74)	3.56 (1.25)	4.17 (0.97)	3.89 (1.09)	4.14 (1.13)	3.64 (1.22)
Surprise (驚き)	36	2.72 (0.97)	4.08 (1.18)	1.58 (0.94)	2.19 (1.04)	2.61 (1.32)	2.11 (1.24)
Unpleasant (不快)	29	1.48 (0.51)	2.59 (1.12)	2.69 (1.49)	3.41 (1.09)	3.14 (1.27)	3.31 (1.39)
Disappointment (落胆)	28	2.00 (1.02)	2.61 (1.55)	2.82 (1.09)	2.82 (1.12)	3.21 (1.34)	2.93 (1.44)
Accomplishment (達成感)	27	4.26 (0.86)	3.26 (1.46)	3.70 (0.91)	3.52 (0.89)	3.78 (0.93)	3.63 (0.84)
Satisfaction (満足)	27	4.22 (0.85)	3.19 (1.30)	3.59 (1.15)	2.93 (1.38)	4.04 (0.81)	3.07 (1.17)
Disgust (嫌悪)	26	1.50 (0.65)	2.81 (1.27)	2.69 (1.05)	3.19 (0.90)	2.69 (0.97)	2.85 (0.97)
Impatience (焦り)	26	2.04 (0.66)	4.12 (1.07)	2.27 (1.15)	2.69 (1.35)	3.50 (1.21)	3.54 (1.27)
Pleasure (快感)	25	4.52 (0.71)	2.76 (1.36)	3.48 (1.33)	3.56 (1.29)	3.80 (1.29)	2.96 (1.40)
Happiness (幸せ)	25	4.76 (0.44)	2.60 (1.58)	3.24 (1.36)	3.36 (1.19)	4.08 (1.04)	3.24 (1.42)
Frustrated (悔しい)	24	1.71 (0.55)	2.75 (1.48)	2.92 (1.21)	3.08 (0.93)	3.25 (1.11)	3.88 (1.23)
Expectation (期待)	24	4.04 (0.55)	3.25 (1.29)	3.29 (1.33)	3.42 (1.02)	3.92 (0.72)	2.67 (1.46)

### The detail of facial movements

**Figure 3:**
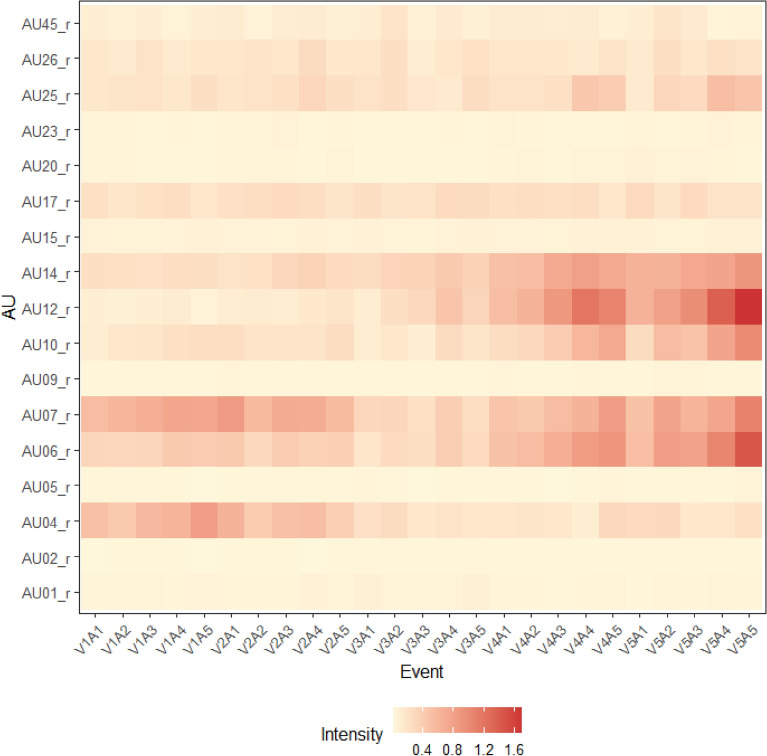
Peak intensities of each facial movement; V represents valence, and A represents arousal.

The facial data for some events are missing due to camera malfunctions and participant problems, although the events themselves were recorded as stated above. The available number of frames was 142,865. When only the peak frame was extracted, there were 1,190 frames. Six events for male and four for female participants were missing expressions. Ultimately, 1,190 data points were available for analysis. Figure 3 shows the facial patterns of all the individual events associated with valence and arousal. Visual inspection revealed that AU4 (lower brow) and AU7 (lid tightener) were strongly expressed during negative events (V1-V2). Positive events (V4-V5) induced AU6 (cheek raiser), AU7, AU10 (upper lip raiser), AU12 (lip corner puller), and AU14 (dimpler), which can be considered strong smiling expressions. The intensity of facial movements may be relatively low in neutral events (V3) compared with the two valenced events. Moreover, in positive events, Fig. 3 indicates that higher arousal was associated with more mouth-opening movements (AU25: lip parts and AU26: jaw drop). In the peak intensity frame, we also checked the correlation between the estimated AUs and appraisal dimensions (Table 4). Compared to the correlations between valence and some facial movements, such as AU12 (lip corner puller: *r* = 0.49), the combinations of all facial movements and other appraisal dimensions were relatively low (|*r*|s < 0.25).

**Table 4:** Correspondence between action units and valence and arousal values and appraisal ratings.

	Valence	Arousal	Predict	Similarity	Important	Control
AU01_r	−0.03	0.02	−0.06	−0.02	0.02	−0.03
AU02_r	0.03	0.00	0.01	0.03	0.01	−0.02
AU04_r	−0.21	0.01	−0.06	−0.11	−0.02	0.06
AU05_r	0.02	0.01	−0.05	−0.02	0.07	−0.09
AU06_r	0.30	0.15	0.07	−0.12	0.21	0.03
AU07_r	0.01	0.08	−0.03	−0.07	0.08	0.04
AU09_r	0.06	−0.03	0.00	0.00	0.02	0.03
AU10_r	0.28	0.18	0.08	−0.09	0.19	0.01
AU12_r	0.49	0.18	0.11	−0.09	0.25	0.00
AU14_r	0.34	0.09	0.07	−0.07	0.16	−0.01
AU15_r	0.00	0.03	0.01	0.00	0.03	−0.03
AU17_r	0.02	−0.02	−0.01	−0.02	0.04	0.03
AU20_r	0.07	−0.03	0.03	0.00	0.04	0.06
AU23_r	0.02	−0.02	0.00	−0.01	0.08	0.03
AU25_r	0.13	0.14	−0.02	−0.08	0.11	0.02
AU26_r	0.02	0.02	−0.02	−0.06	0.01	0.01
AU45_r	0.03	−0.04	0.00	0.00	0.00	−0.05

A hierarchical linear regression model examined the relationship between valence/arousal and the AUs. Consistent with our predictions, the result indicated that the valence values significantly predicted the intensity of AU4 (brow lowerer) negatively (*β* = −0.11, *t* = 6.38, *p* < 0.001) and that of AU12 (lip corner puller) positively (*β* = 0.28, *t* = 12.64, *p* < 0.001). Besides, the arousal values significantly predicted the intensity scores of AU 12 (*β* = 0.10, *t* = 9.19, *p* < 0.001). Furthermore, post-hoc sensitivity power analysis using the simr package^75^ indicated that the current sample size (i.e., *N* = 1190) was sufficient to detect all coefficients in the hierarchical linear regression models with a significance level of α = 0.05 and 99% power.

To explore the new relationship between arousal and the AUs, we also used the Bayesian Lasso regression. Action Units 12 (lip corner puller) and 25 (opening the mouth) were found to predict arousal (*β*s = 0.13, 95% Credible Intervals [0.01, 0.26] and 0.08, 95% Credible Intervals [0.00, 0.17]). However, none of the other predictors predicted arousal performance, resulting from 95% CIs that included zero.

We confirmed the dynamics of the facial expressions obtained in this database by applying non-negative matrix factorization to reduce dimensionality and extract spatiotemporal features ^76^. This approach can identify dynamic facial patterns^77–79^. The factorization rank was determined using cophenetic coefficients^80^ and the dispersion index^81^. Information on factorization rank is available on the Gakunin RDM (https://dmsgrdm.riken.jp:5000/uphvb/).

**Figure 4:**
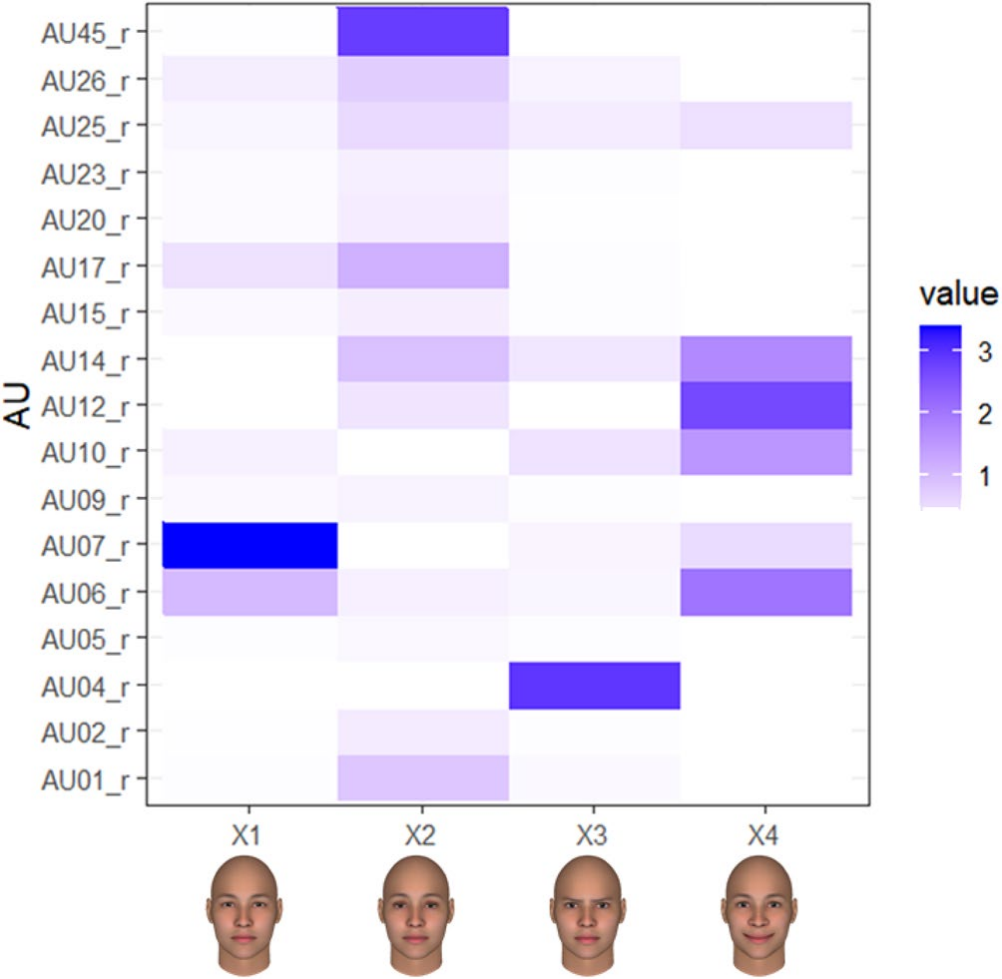
Heatmap of each component’s loadings for facial expressions of all events. Value colors represent each facial movement’s contribution to component scores.

Figure 4 displays the AU profiles of the top four components. We interpreted Component 1 as a Duchenne marker (AU6, 7), Component 2 as blinking and other facial movements (AU1, 14, 17, 45), Component 3 as a lower brow (AU4), and Component 4 as smiling (AU6, 10, 12, 14) by visually inspecting the relative contribution of each AU to the independent components. These results were also consistent with the peak intensities of each facial movement (Fig. 3).

**Figure 5:**
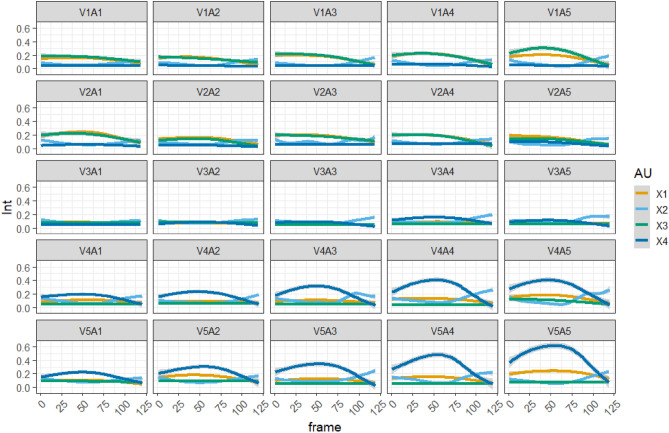
Temporal changes in the four components for the facial expressions of 25 events.

Figure 5 lists how the spatial components changed over time for each valence and arousal combination. Visual inspection of component 1 (Duchenne marker) revealed that negative (V1-V2) and positive (V4-V5) events showed larger movements (e.g., V1A1 and V5A5). This result is consistent with the finding that eye constriction is systematically associated with the facial expressions of negative and positive emotions^82^. Component 2 (blinking and other facial movements) can be interpreted as the relaxation movement of tension associated with the expression of deliberate facial manipulation or noise unrelated to the main emotional expression because this movement increases during the offset duration (frames = 91–120) after the peak duration (frames = 31–90). For Component 3 (lower brow), negative expressions (V1–V2) produced more intense facial changes than other expressions (V3–V5). Component 4 (smiling) occurred more frequently during positive events (V4–V5) than others (V1–V3).

In summary, blinking and other facial movements, such as raising the inner eyebrow and chin, were (i.e., Component 2) peculiar to the offset of deliberate facial expressions in naive Japanese participants. More interestingly, the results clarified that smiling is related to the positive (Component 4), lowering of eyebrows is related to the negative (Component 3), and eye constriction (Component 1) corresponds to both values.

**Figure 6:**
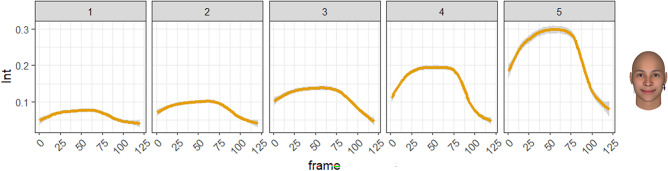
Temporal changes in the smiling pattern for the appraisal check of importance.

As a supplementary analysis and an example of the potential uses of the database, it may be useful to visualize dynamic changes rather than correlations in the peak frame (Table 4) as the relationship between one appraisal dimension and one facial pattern. According to Scherer’s theory, the appraisals (and the corresponding AUs) appear sequencetially. Figure 6 shows the relationship between one appraisal dimension (important) and one component (AU6, 10, 12, 14). This indicates that as the appraisal of the importance of an event increase, more smiles are seen in response to the event.

## Discussion

This study developed a new facial database with expresser annotations such as individualized emotional events, appraisal checks, and free description labels with multi-angle and depth information. The results (Table 3) indicate that the words for each event had few matches, implying that the database has a large variance in emotional events. A database with various events and individual evaluations can be verified for academic purposes. For example, researchers can investigate issues such as the typical elements of events labeled as anger and the appraisal components that constitute them in a data-driven manner or as a starting point.

According to the analysis of front-view facial expressions, facial movements related to pleasant and unpleasant valences were observed. For example, lowering the brow was related to a negative valence, whereas pulling the lip corner was related to a positive valence. These results are consistent with previous findings investigating the coherence between valence and facial muscle electrical activity. Moreover, the Bayesian lasso analysis reported that mouth movements such as AU12 (pulling the lip corner) and AU25 (opening the mouth) were also associated with arousal. Opening the mouth has been shown to increase arousal attribution from observers^40^, which corresponds to ratings on the part of the expressers. This contributed to understanding the relationship between specific facial action and arousal. In addition to the data provided here, we are currently performing manual facial action coding by certified FACS coders. We will open the annotation data in the future (now, data for 8 people is already annotated and available in the same database. There are 29 types of manually annotated facial actions). In recent years, amidst the controversy about emotion^83^, there have been increasing efforts to extract facial movements^84^. The opening of databases, including manual FACS annotations that include in-depth information, can prime how research in affective computing can be further developed.

While this study provides a new facial database on emotions, certain limitations exist. First, the number of participants was small, given the diversity of facial movements and emotional events. In particular, the database only includes recordings of Japanese participants, which may limit its generalizability to other populations. Future research using similar environments, as represented in Fig. 2, will create additional databases for young and older adult participants and extend to other cultures or ethnicities beyond the Japanese population. Second, this study dealt with only facial responses to emotional expression. However, other aspects such as vocal or physiological responses would be important for understanding emotional communication^85–87^. Expansion of those modalities could provide a useful database to understand emotion further. Finally, we did not investigate how depth or infrared information can be used, and the lighting conditions do not influence this information compared to 2D color images. This database will be an important foundation for developing a robust sensing system for facial movements in room conditions. Using these databases, we provide an internal state estimation algorithm via an Application Programming Interface combined with smartphones and other devices. Furthermore, we would like to utilize this technology to develop solutions for people with difficulty communicating.

The database, including the expressers’ events, labels, and appraisal checking intensity, is available as a RIKEN facial expression database for academic purposes. The notable features of this database are as follows: (a) availability of multiple theoretical views for emotion (valence and arousal, appraisal dimensions, and free emotion label), (b) variety of events, and (c) rich information taken from 10 multi-angle and depth cameras.

## Data Availability

The RIKEN facial expression database is freely available to the research community (https://dmsgrdm.riken.jp:5000/uphvb/). An End User License Agreement (EULA) must be produced to access the database.
